# Impaired AT2 to AT1 cell transition in PM2.5-induced mouse model of chronic obstructive pulmonary disease

**DOI:** 10.1186/s12931-022-01996-w

**Published:** 2022-03-25

**Authors:** Hongjiao Yu, Yingnan Lin, Yue Zhong, Xiaolan Guo, Yuyin Lin, Siqi Yang, Jinglin Liu, Xinran Xie, Yaowei Sun, Dong Wang, Bing Li, Pixin Ran, Jianwei Dai

**Affiliations:** 1 Guangzhou Medical University-Guangzhou Institute of Biomedicine and Health (GMU-GIBH) Joint School of Life Sciences, Guangzhou Medical University, Guangzhou, 510000 People’s Republic of China; 2 State Key Lab of Respiratory Disease, National Clinical Research Center for Respiratory Disease, Guangzhou Institute of Respiratory Disease, The First Affiliated Hospital of Guangzhou Medical University, Guangzhou, 510120 People’s Republic of China; 3 The Sixth Affiliated Hospital of Guangzhou Medical University, Qingyuan People’s Hospital, Guangzhou Medical University, Qingyuan, 511500 People’s Republic of China

**Keywords:** PM2.5, COPD, Alveolar epithelium, AT2-to-AT1 transition

## Abstract

**Background:**

Particular matter 2.5 (PM2.5) is one of the most important air pollutant, and it is positively associated with the development of chronic obstructive pulmonary disease (COPD). However, the precise underlying mechanisms through which PM2.5 promotes the development of COPD remains largely unknown.

**Methods:**

Mouse alveolar destruction were determined by histological analysis of lung tissues and lung function test. Alveolar type II cells (AT2) to alveolar type I cells (AT1) transition in PM2.5-induced COPD mouse model was confirmed via immunofluorescence staining and qPCR analysis. The differentially expressed genes in PM2.5-induced COPD mouse model were identified by RNA-sequencing of alveolar epithelial organoids and generated by bioinformatics analysis.

**Results:**

In this study, we found that 6 months exposure of PM2.5 induced a significantly decreased pulmonary compliance and resulted in pulmonary emphysema in mice. We showed that PM2.5 exposure significantly reduced the AT2 to AT1 cell transition in vitro and in vivo. In addition, we found a reduced expression of the intermediate AT2-AT1 cell process marker claudin 4 (CLDN4) at day 4 of differentiation in mouse alveolar organoids treated with PM2.5, suggesting that PM2.5 exposure inhibited AT2 cells from entering the transdifferentiation process. RNA-sequencing of mouse alveolar organoids showed that several key signaling pathways that involved in the AT2 to AT1 cell transition were significantly altered including the Wnt signaling, MAPK signaling and signaling pathways regulating pluripotency of stem cells following PM2.5 exposure.

**Conclusions:**

In summary, these data demonstrate a critical role of AT2 to AT1 cell transition in PM2.5-induced COPD mouse model and reveal the signaling pathways that potentially regulate AT2 to AT1 cell transition during this process. Our findings therefore advance the current knowledge of PM2.5-induced COPD and may lead to a novel therapeutic strategy to treat this disease.

**Supplementary Information:**

The online version contains supplementary material available at 10.1186/s12931-022-01996-w.

## Introduction

Chronic obstructive pulmonary disease (COPD) is one of the most common form of chronic lung respiratory diseases, and it is characterized by accelerated decline of lung function and irreversible airway obstruction due to chronic bronchitis, small airway remodeling and emphysema [1]. COPD is a life-threatening disease and one of the leading causes of mortality and morbidity in the world [2]. It is currently recognized that COPD is primarily caused by cigarette smoke, air pollution such as particular matter 2.5 (PM2.5), environmental factors and aging [3]. Over the past decades, extensive efforts have been paid to explore the pathogenic mechanisms underlying COPD. Several mechanisms have been reported to paly crucial roles in the development and progression of COPD, including inflammation, imbalanced activity of proteases and anti-protease, apoptosis of epithelial and endothelial cells, oxidative stress and cellular senescence [4, 5]. However, the precise molecular and cellular mechanisms, and the cues that drive the development of COPD remain to be fully elucidated.

The alveolar epithelia of lungs consist of both alveolar type I cells (AT1) and alveolar type II cells (AT2). AT1 cells that are critical for gas exchange in the lungs show a flattened squamous shape, which cover about 95% of the alveolar surface area [6]. Injury of AT1 cells induced by PM2.5, cigarette smoke, pathogens or release of inflammatory cytokines may lead to life-threatening disease such as COPD and pneumonia [7]. On the other hand, AT2 cells show similar in number to AT1 cells, but only occupy 5% of the alveolar surface area. AT2 cells produce surfactant, and protect lungs against infections to maintain lung homeostasis [8]. Interestingly, AT2 cells have been shown to act as stem cells to repair the damaged epithelium depending on their capacity for self-renewal and differentiation to AT1 cells [6, 9]. Previous studies have reported several key molecular signaling pathways that are required for AT2 to AT1 transition and the alveolar epithelium repair such as the Notch signaling, Transforming Growth factor (TGF)-signaling and Wnt signaling [10–12]. Emerging evidence supports that impaired alveolar epithelial homeostasis plays a crucial role in the pathogenesis of COPD [13–15].

Cigarette smoking is considered as one of key risk factor for the development of COPD, which leads to airway inflammation and abnormal tissue repair in the lungs [16]. However, only a small proportion of smokers (10–25%) eventually develop COPD [17], and approximately 25% of COPD patients are non-smokers [17]. These results suggest that other factors may also contribute significantly to the development and progression of COPD. Interestingly, previous epidemiologic studies have demonstrated that increased air pollution is associated with COPD [18–20]. Ambient particulate matter with an aerodynamic diameter of 2.5 μm or less is considered as one of the most important air pollutant, which contains various toxic chemicals. Previous studies have reported a positive association between PM2.5 and COPD hospitalization, morbidity and mortality, as well as respiratory function and symptoms of COPD patients [20–23]. In addition, PM2.5 exposure has been reported to induce pulmonary inflammation, decrease lung function, and cause emphysematous changes [3]. PM2.5 exposure may promote COPD via regulation of the Notch signaling pathway [24], which is required for AT2 to AT1 transition during the alveolar epithelium repair [12]. However, whether AT2 to AT1 transition directly contribute to PM2.5-induced COPD and the underlying mechanisms remain largely unknown.

The present study aims to investigate the effects of PM2.5 exposure on AT2 to AT1 transition and alveolar epithelium repair using COPD animal models, and explore the underlying mechanisms through which PM2.5 regulate AT2 to AT1 transition during COPD.

## Methods

### Preparation of PM2.5

As previously described [25], PM2.5 was collected from the burning of China fire in a conventional Chinese wood stove with a high-volume sampling machine (TE-6070, Tisch, USA) equipped with a PM2.5 selective-inlet head (1.13 m^3^ /min). PM2.5 was collected on glass fiber membrane filters with a 1.6-μm pore size and a 406-cm^2^ sampling area. Particles were sampled during high-temperature combustion achieved with moderate air supply. The amount of PM2.5 was defined as the weight increase for each filter. The filters were soaked in dimethyl sulfoxide (DMSO), and the solutions were then filtered through a 5-μm needle filter. The supernatant was collected, and the particles recovered from different filters were pooled to ensure a homogenous batch of particles. The 100 mg/mL PM2.5 stock solution was stored at − 20 °C until use. Before used for experiments, the PM2.5 solution was sonicated 3 times, 25 s per time.

### Mice

It has been reported that women have a higher risk to develop COPD than men since women are more frequently exposed to indoor pollutants produced by biomass fuels burning during household activities [26, 27]. Hence, we used female mice in this study. PM2.5 exposure experiments were performed as previously described [28], eight to ten weeks old female C57BL/6 mice were housed in rooms maintained at a constant temperature and humidity with a 12-h light cycle and were allowed to access rodent laboratory chow and water ad libitum. All mouse protocols were approved by the Institutional Animal Care and Use Committee at the Guangzhou Medical University. Mice were exposed to PM2.5 from bio-mass fuels or fresh air for 6 h every day continuously for 6 months. After that mouse lung function was tested. Mice were then sacrificed and lungs were collected for further analysis. For mouse alveolar organoids culture experiments, 8 weeks old female C57BL/6 mice were fed in rooms maintained at a constant temperature and humidity under 12:12 light/dark cycles and were allowed to access rodent laboratory chow and water ad libitum.

### Lung function test

After intraperitoneal anesthesia with 0.3% pentobarbital sodium anesthetic, mice were placed on the operating table and connected to Buxco Pulmonary Function Test (BUXCO, PFT) by tracheal intubation for detection of Boyle’s Law Functional Residual Capacity (FRC), Fast Flow Volume (FV), Quasi-static Pressure Volume (PV) and Resistance and Compliance (RC).

### Lung cell isolation and AT2 sorting

Tissues were disrupted and cells were collected after lungs were inflated and digested with a cell digestion solution (DMEM/F12 medium with 2 mg/mL collagenase type I, Solarbio, C8140) for 1 h at 37 °C with frequent agitation. Digestion was terminated by addition of DMEM/F12 medium with 10% FBS (ExCell Bio, FSP500) and cells were resuspended in 1X RBC Lysis Buffer (Invitrogen, 00-4333-57) for 5 min to lyse red blood cells. After centrifugation at 500 g for 10 min at 4 °C, remaining cells were resuspended in Magnetic Bead Buffer and incubated with CD45 microbeads (Mitenyi Biotech, 130-052-301) for 20 min at 4 °C. After that incubated cells were sorted by LS column (Mitenyi Biotech, 130-042-401) and washed by adding 9 mL Magnetic Bead Buffer. CD45^+^ cells were removed by 10 min centrifugation at 500 g and the remaining cells were incubated with CD326 microbeads (Mitenyi Biotech, 130-105-958) for 20 min at 4 °C. After incubation, cells were sorted by MS column (Mitenyi Biotech, 130-042-201) and washed by adding 3 mL Magnetic Bead Buffer. AT2 cells were isolated with negative selection of CD45 and positive selection of CD326 according to previous studies [29, 30], the purity of AT2 cells was validated by flow cytometry analysis (Additional file 4: Fig S2).

### Organoid culture

The organoids were cultured as previously described [31, 32]. Briefly, freshly sorted cells were resuspended in serum free DMEM/F12 Basic Medium (Gibco, C11330500BT) supplemented with 1X penicillin/streptomycin and 1X B-27™ Supplement (Gibco, 17504044), and mixed with equal amount of growth factor-reduced Matrigel (Corning, 356234). DMEM/F12 Basic Medium/Matrigel containing 5 × 10^3^ CD45^−^CD326^+^ AT2 cells and 5 × 10^4^ CD45^−^CD326^−^ non-AT2 cells were seeded in 12-well plates for 7–10 days in Alveolar Maintain Medium with 50 ng/mL EGF (MedChemExpress, HY-P7076), 10 ng/mL FGF10 (MedChemExpress, HY-P7170), 10 ng/mL Noggin (MedChemExpress, HY-P7051A), 10 μmol/L SB-431542 (MedChemExpress, HY-10431) and 3 μmol/L CHIR99021 (MedChemExpress, HY-10182), followed by 4 days treatment with 10 ng/mL IL-1β (MedChemExpress, HY-P7073A) and 10 μmol/L Y-27632 (MedChemExpress, HY-10071). Medium was changed every other day. After 7–10 days’ treatment with Alveolar Maintain Medium, cell medium was switched to Alveolar Differentiation Medium which contains 10% of FBS and 10 μmol/L XAV-939 (MedChemExpress, HY-15147), and cells were cultured for an additional 7 days.

### Organoids culture with PM2.5

To investigate the influences of PM2.5 long term exposure in alveolar regeneration, PM2.5 was diluted in Alveolar Maintain Medium and Alveolar Differentiation Medium to 50 μg/mL and added to the alveolar organoids for PM2.5 exposure group. The CONTROL group was added with Alveolar Maintain Medium and Alveolar Differentiation Medium without PM2.5. Medium containing 10 μM ROCK inhibitor Y27632 was changed every other day.

### Histology and immunofluorescence

For histological analysis, lung tissues were fixed overnight with 4% paraformaldehyde (biosharp, BL539A) at 4 °C. Paraffin sections were generated and used for Haemotoxylin & Eosin (H&E) staining. For Immunofluorescence study, lung sections were fixed with 4% paraformaldehyde for 20 min at room temperature and permeabilised with 0.3% Triton X-100 for 10 min. Then lung sections were blocked in 10% BSA/0.1% Triton X-100/PBS for 1 h in room temperature. After incubation with blocking solution, the tissue sections were incubated with rabbit polyclonal antibody against Pro-surfactant Protein C (1:1000, Millipore, AB3786), mouse polyclonal antibody against HOPX (1:100, Santa Cruz Bio, sc-398703), mouse polyclonal antibody against PIEZO 1 (1:100, Signalway, #48073), rat polyclonal antibody against Ki67 (1:100, Invitrogen, 14-5698-95), rabbit polyclonal antibody against Claudin 4 (1:100, Invitrogen, 36-4800), or cleaved caspase-3 antibody (1:200, Cell Signaling Technology, #9661) in 4 °C for overnight. After removing the primary antibody, the tissue sections were incubated with donkey anti- rabbit IgG Alexa Fluor 594, donkey anti- rabbit IgG Alexa Fluor 647, donkey anti- mouse IgG Alexa Fluor 488, or donkey anti- rat IgG Alexa Fluor 594 labeled secondary antibody (1:1000, Invitrogen) for 1 h in room temperature. For nuclear staining, the sections were incubated with DAPI (1:1000, Biofroxx, 1155MG010) in PBS for 8 min at room temperature.

### RNA preparation and RT-PCR

Total RNA was extracted from organoids using NucleoZOL (MNG, 740404.200). cDNA was obtained using Evo M-MLV RT Kit (Accurate Biology, AG11707). Relative gene expression was measured by real-time PCR using SYBR Green Premix Pro Taq HS qPCR Kit (Accurate Biology, AG11701). Gene expression was quantified using the ΔΔC_t_ method and normalized by *Gapdh* using the following primers:

*Cdc25c* (5′-ACAGGACCTATCCCACCTGC-3′ and 5′-TTAGGTTTGCCGAGTCGT GG-3′); *Cyclin B1* (5′-TGCGCCTGCAGAAGAGTATC-3′ and 5′-CCAGTCACTTCA CGACCCTG-3′); *BMPR1B* (5′-GCTGCACAGAAAGGAATGAGTG-3′ and 5′-GGG CCCATCAACAAAATCTCTG-3′); *AXL* (5′-TGATAACACCCAGACCCAGG-3′ and 5′-TG ACTCCCTTGGCATTGTGG-3′); *VEGFA* (5′-GTCCGATTGAGACCCTGGTG -3′ and 5′-TTGACCCTTTCCCTTTCCTCG-3′); *KLF4* (5′-CCAGTATACATTCCGC CACAG-3′ and 5′-TCTGGGCTTCCTTTGCTAAC-3′); *BRAF* (5′-ACAGAATTCAG GATGGAGAGAAGA-3′ and 5′-GTTGTGTGTTGTAAGTGGGACA-3′); *DLL1* (5′-T TGCTTCAATGGAGGACGAT-3′ and 5′-ACACTTGGCACCGTTAGAACA-3′); *ROR2* (5′-ATCGACACCTTGGGACAACC-3′ and 5′-AGTGCAGGATTGCCGTCTG -3′); *SIK1* (5′-CTACAACCACTTTGCCGCCAT-3′ and 5′-AGGGGGAATAATAAGG GCTGAAG-3′); *SFRP4* (5′-ACCTGAGCAAAAACTACAGCTATG-3′ and 5′-CTAC CACAGTTGTGACCTCATTG-3′); *ITGAV* (5′-GGTGACTCAATCTGTGACCTTCA GC -3′ and 5′-CACAAATCAAGGATGACCAAACTGAG-3′); *GLI1* (5′-CGCCCCG ACG GAGGTCTCT-3′ and 5′-GCTGGCCGTCCCAACTGCTT-3′).

### RNA sequencing

Total RNA was isolated from the lung tissues using NucleoZOL (MNG, 740404.200). Quality and quantity of isolated RNA were checked and measured with NanoDrop ND-2000 (Thermo Fisher Scientific, Waltham, MA, USA). rRNAs were removed from isolated RNA using Ribo-Zero rRNA Removal Kits (Illumina, USA) following the manufacturer's instructions. RNA libraries were constructed using rRNA-depleted RNAs with TruSeq Stranded Total RNA Library Prep Kit (Illumina, USA) according to the manufacturer’s instructions. Libraries were controlled for quality and quantified using the BioAnalyzer 2100 system (Agilent Technologies, USA). 10 pM libraries were denatured as single-stranded DNA molecules, captured on Illumina flow cells, amplified in situ as clusters and finally sequenced for 150 cycles on Illumina HiSeq Sequencer according to the manufacturer’s instructions. The high quality reads were aligned to the musculus reference genome (UCSC mm10) with hisat2 software. Then, guided by the Ensembl gtf gene annotation file, cuffdiff software (part of cufflinks) was used to get the Fragments Per Kilobase Million (FPKM) as the expression profiles of mRNA, and fold change and p-value were calculated based on FPKM, differentially expressed mRNA were identified.

### GO analysis and KEGG analysis

Visualization of differentially expressed genes, Venn diagram analysis, Gene Ontology analysis and KEGG analysis were generated in the website HIPLOT (https://hiplot.com.cn/basic). The protein–protein interaction network analysis was generated by STRING (https://cn.string-db.org/). The preliminary data analysis was generated using the Database for Annotation, Visualization and Integrated Discovery (DAVID, https://david.ncifcrf.gov/).

### Quantification and statistics

All quantifications of H&E staining and Immunofluorescence experiments were performed using Image J. Statistical analyses were performed using GraphPad Prism 6. Each experiment was repeated at least 3 times. Biological replicates were sex and age matched whenever possible.

## Results

### PM2.5 induced pulmonary emphysema in the alveoli of mice

**Fig. 1:**
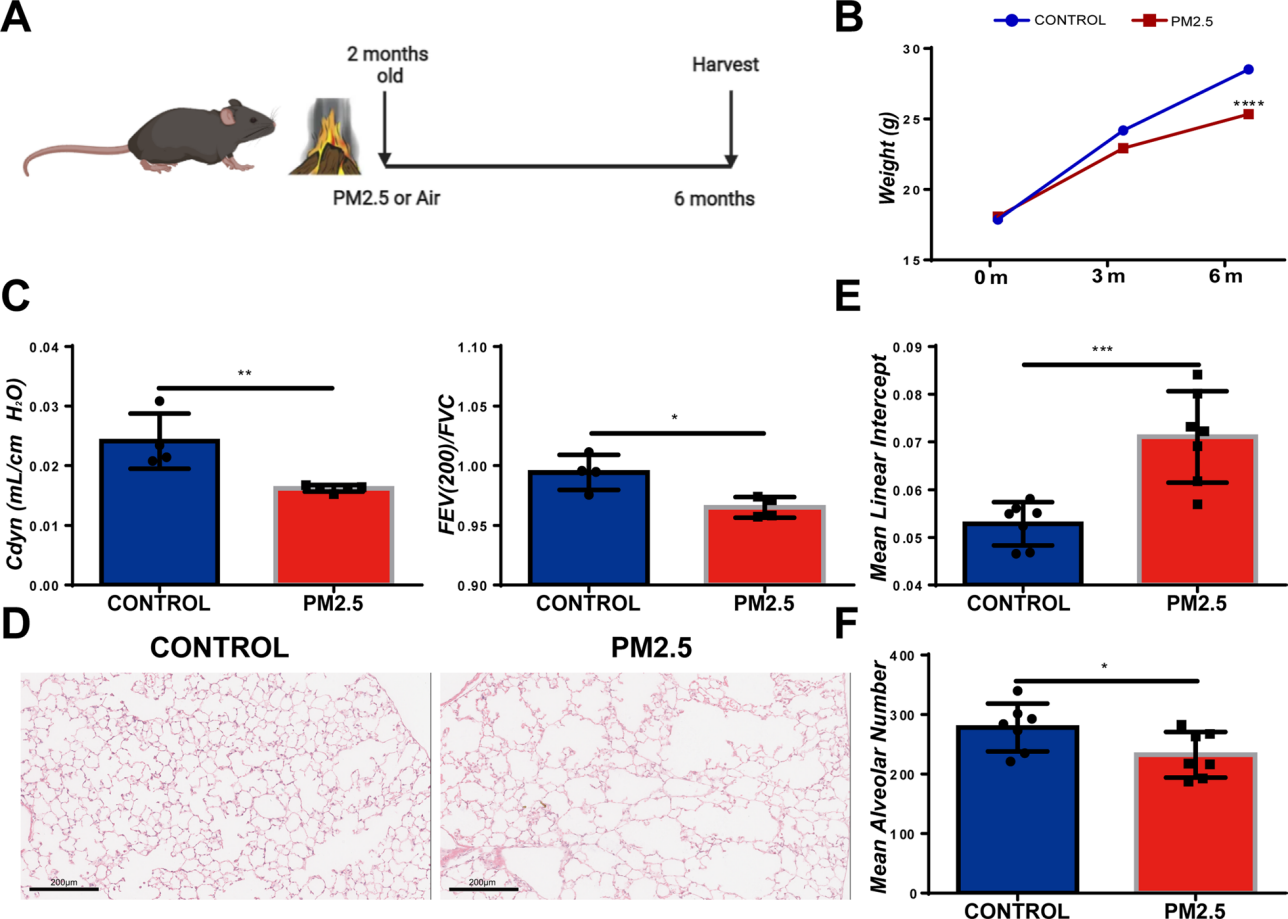
PM2.5 causes abnormal respiratory system in mice. C57BL/6 mice were exposed to biomass fuel PM2.5 for six months and analyzed on indicated days. Lung function was examined in anesthetized mice using DSI animal pulmonary function test machine. **A** Scheme for biomass fuel PM2.5 long-term exposure system modeling. **B** Dynamic weight changes in control and long-term PM2.5-exposed mice. **C** Lung function test of CONTROL and PM2.5 exposure group. Cdyn: dynamic lung compliance. FEV (200)/FVC: ratio of forced expiratory volume to forced vital capacity at 200 ms. PM2.5 exposure reduced dynamic Lung Compliance and FEV (200)/FVC. **D** HE staining of CONTROL and PM2.5-exposed mice. Scale bar = 200 μm. **E** Mean Alveolar Number (MAN) was counted in CONTROL and PM2.5-exposed mice. **F** Mean Linear Intercept (MLI) was examined in CONTROL and PM2.5-exposed mice. *p < 0.05, **p < 0.01, ***p < 0.001, ****p < 0.0001. Comparisons between two groups (CONTROL vs PM2.5 exposure) were analyzed by unpaired t test. Data presented are mean ± SD. n ≥ 4

In order to evaluate the effects of PM2.5 exposure on the respiratory system of mice, we constructed a biomass fuel PM2.5 long-term exposure system, as shown in Fig. 1A. PM2.5 exposure caused a significant decrease in the body weight (Fig. 1B) and decreased pulmonary compliance in mice. Consistently, forced pulmonary ventilation at 200 ms was significantly reduced in these mice (Fig. 1C). Mouse pulmonary emphysema was observed after long-term exposure to PM2.5 (Fig. 1D), with an increase in the mean alveolar linear intercept (Fig. 1E) and a decrease in the mean number of alveoli (Fig. 1F). These data revealed that long-term PM2.5 exposure induced a significant damage to alveolar structure.

### PM2.5 exposure reduced AT1/AT2 ratio and enhanced the proliferation ability of AT2

**Fig. 2:**
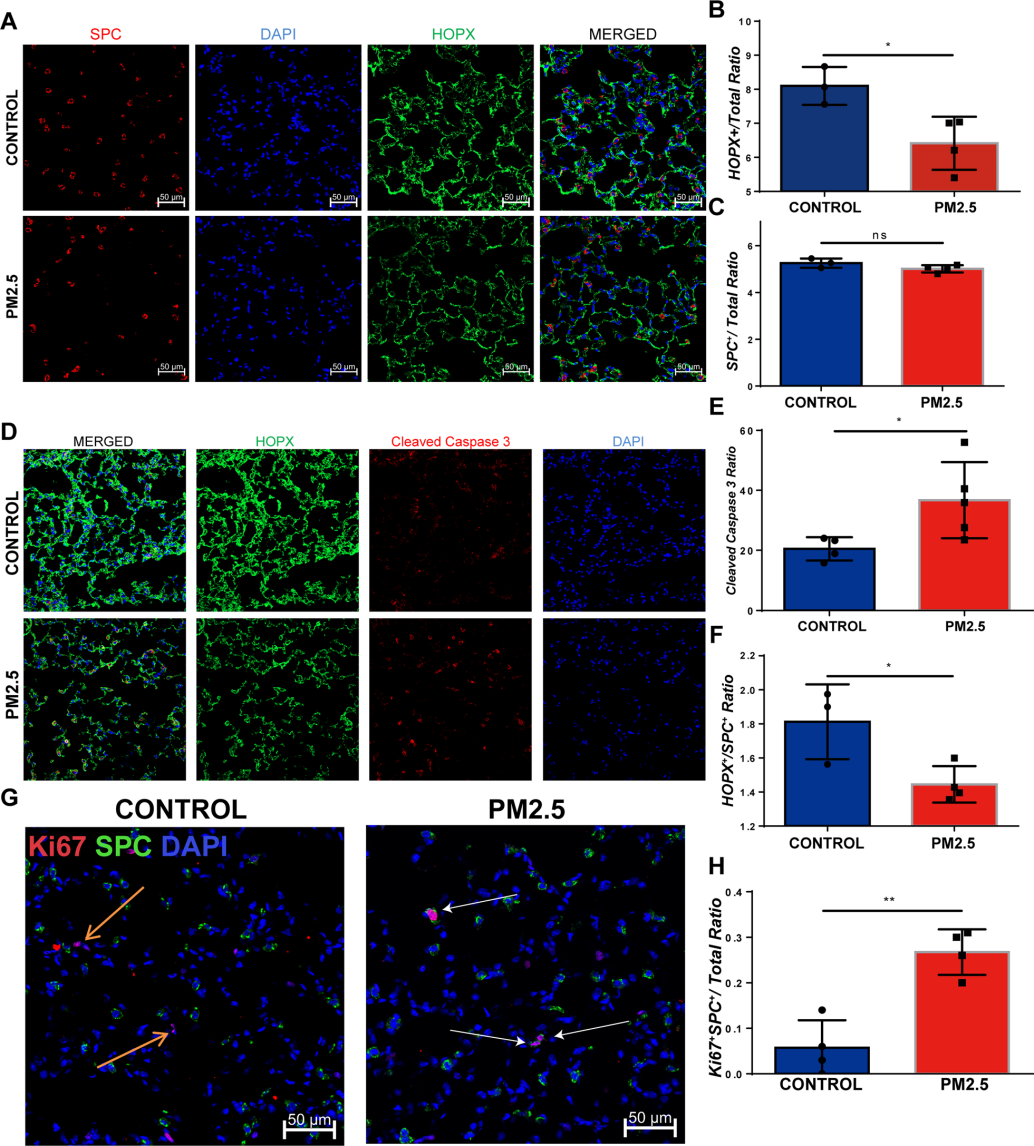
PM2.5 induced differential m^6^A modification in COPD. PM2.5 exposure reduced AT1/AT2 ratio and unaffected the proliferation ability of AT2. **A** Immunofluorescence staining of CONTROL and PM2.5-exposed lung tissues for AT1 and AT2. AT1 cells were marked with HOPX and AT2 cells were marked with SPC. Scale bar = 50 μm. **B** Quantitative of AT1/total cell ratio per 20 × field. **C** Quantitative of AT2/total cell ratio per 20 × field. **D** Immunofluorescence staining of CONTROL and PM2.5-exposed lung tissues for AT1 and Cleaved Caspase 3. Scale bar = 50 μm **E** Quantitative analysis of the ratio of Cleaved Caspase 3 positive AT1 cells vs total AT1 cells per 20 × field. **F** Quantitative of AT1/AT2 ratio per 20 × field. **G** Immunofluorescence SPC and Ki67 in CONTROL and PM2.5-exposed lung tissues. Ki67 is a marker for cells in proliferating phase. The orange arrows indicate the Ki67 staining and the white arrows indicate a co-localization of SPC and Ki67. Scale bar = 50 μm. **H** Quantitative of SPC^+^Ki67^+^ cell number per 20 × field. *p < 0.05, **p < 0.01. Comparisons between two groups (CONTROL vs PM2.5 exposure) were analyzed by unpaired t test. Data presented are means ± SD. The experiments were repeated at least three times (n ≥ 3)

We next determined whether PM2.5 exposure affected the function of alveolar epithelial cells. After long-term PM2.5 exposure, the number of AT1 cells was decreased and the ratio of AT1/total cells was significantly reduced (Fig. 2A and B), the number of AT2 cells was also reduced but the ratio of AT2/total cells has no significant change (Fig. 2A and C). We further showed that long-term PM2.5 exposure induced a significantly increased apoptosis in AT1 cells (Fig. 2D and E). Consistently, PM2.5 exposure resulted in a lower AT1/AT2 ratio (Fig. 2F). The alveolar repair process is mainly accomplished by AT2 proliferation and the transdifferentiation of AT2 into AT1. The reduction of AT1 cells and the AT1/AT2 ratio suggests that PM2.5 exposure affects the reparative function of AT2 cells. We found that PM2.5 exposure increased the expression of the proliferation marker Ki67 and the number of proliferating AT2 cells (Fig. 2G and H).

### PM2.5 probably influence the repair of alveolar damage by affecting AT2 to AT1 transition.

**Fig. 3:**
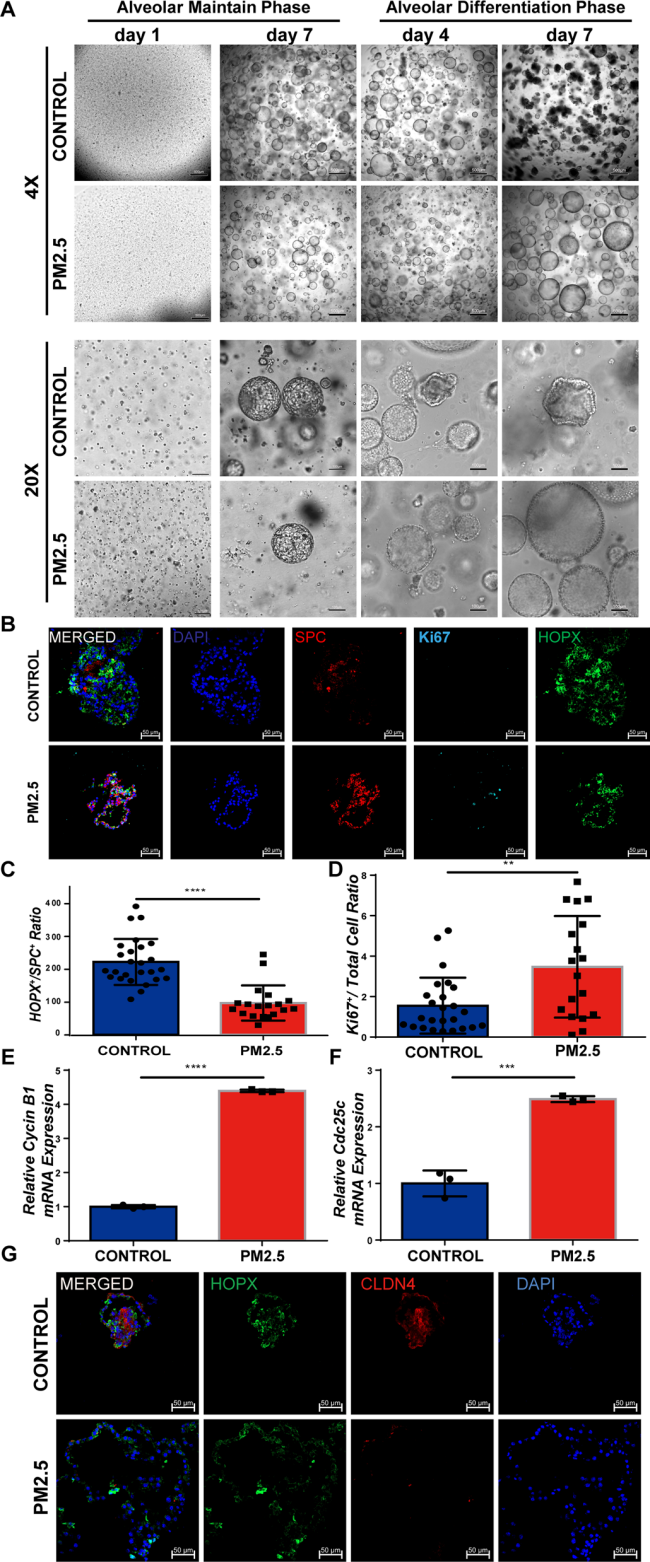
PM2.5 is likely to influence the repair of alveolar damage by affecting the transdifferentiation of AT2 to AT1. **A** The culture process of mouse alveolar organoids. Scale bar = 500 μm or 100 μm as indicated. **B** Immunofluorescence staining of alveolar organoids in CONTROL group and PM2.5-exposed alveolar organoids for HOPX, SPC and Ki67 in the alveolar differentiation phase day 4. Scale bar = 50 μm. **C** Quantitative of AT1/AT2 ratio per 20 × field. **D** Quantitative of Ki67^+^/total ratio per 20 × field. **E**, **F** The mRNA expression of cell cycle related genes Cdc25c and Cyclin B1 in the alveolar differentiation phase day 4. **G** Immunofluorescence staining of intermediate AT2-AT1 cell process marker CLDN4 in the alveolar differentiation phase day 4. Scale bar = 50 μm. **p < 0.01, ***p < 0.001, ****p < 0.0001. Comparisons between two groups (CONTROL vs PM2.5 exposure) were analyzed by unpaired t test. Data presented are means ± SD. The experiments were repeated at least three times (n ≥ 3)

We further evaluated whether PM2.5 exposure affects AT2 to AT1 transition during the alveolar damage and repair process using mouse alveolar organoids culture in vitro (Additional file 4: Fig. S1A). PM2.5 exposure reduced organoid forming efficiency in alveolar maintain phase, but the pattern of organoid had no significant difference (Fig. 3A). Interestingly, alveolar organoids in the CONTROL group were shrinking while the PM2.5-exposed organoids were still increasing in alveolar differentiation phase (Fig. 3A). We detected an increased number of AT2 cells (Additional file 4: Fig. S1B) and a decreased number of AT1 cell (Fig. 3B, Additional file 4: Fig. S1C and S1D) in the PM2.5-exposed alveolar organoids in its differentiation phase. Consistently, the ratio of AT1/AT2 decreased (Fig. 3C). Meanwhile, number of cells in proliferating phase was significantly increased (Fig. 3B and D) and the expression of cycle-related genes Cdc25c and Cyclin B1 were also increased significantly (Fig. 3E and F), indicating PM2.5 exposure enhances AT2 cell proliferation. However, the expression of the intermediate AT2-AT1 cell process marker claudin 4 (CLDN4) was reduced in alveolar differentiation phase day 4 (Fig. 3G), suggesting that PM2.5 exposure inhibits AT2 cells from entering the transdifferentiation process. These data indicate that PM2.5 exposure induced damage to alveolar organoids and led to an enhanced AT2 proliferation and an impaired repair processes.

### The abnormal AT2 damage and repair process caused by PM2.5 exposure may be related to the down-regulation of cell differentiation genes

**Fig. 4:**
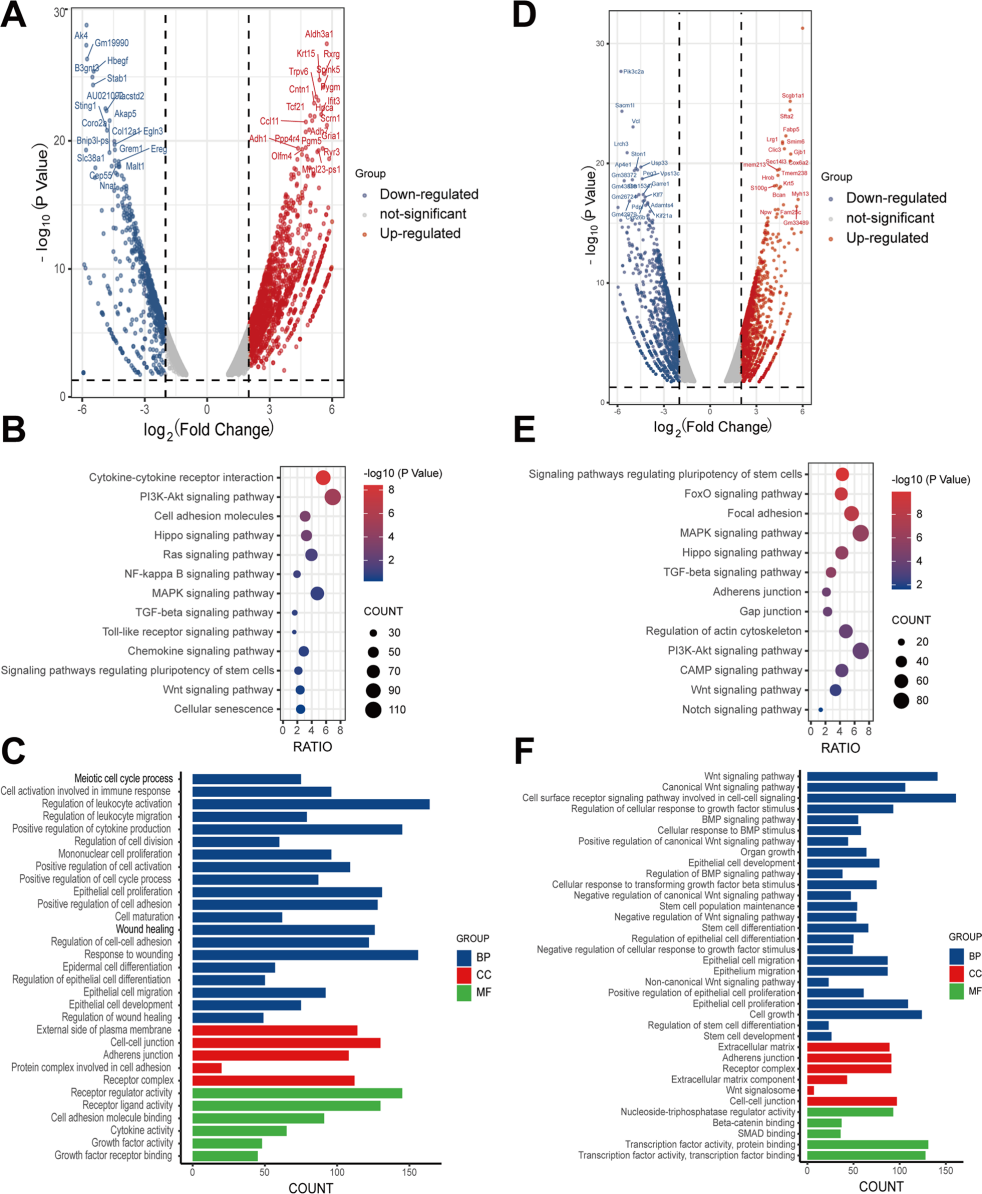
AT2 damage and repair induced by PM2.5 exposure may be related to the down-regulation of cell differentiation genes. **A** Visualization of differentially expressed genes in alveolar maintaining phase. The red dots represent up-regulated differentially expressed genes, the blue dots represent down-regulated differentially expression genes and the grey dots represent genes with no significant change. **B** Pathway analysis of down-regulated genes in alveolar maintaining phase. **C** GO analysis of down-regulated genes in alveolar maintaining phase. **D** Visualization of differentially expressed genes in alveolar differentiation phase. The red dots represent up-regulated differentially expressed genes, the blue dots represent down-regulated differentially expression genes and the grey dots represent genes with no significant change. **E** Pathway analysis of down-regulated genes in alveolar differentiation phase. **F** GO analysis of down-regulated genes in alveolar differentiation phase

**Fig. 5:**
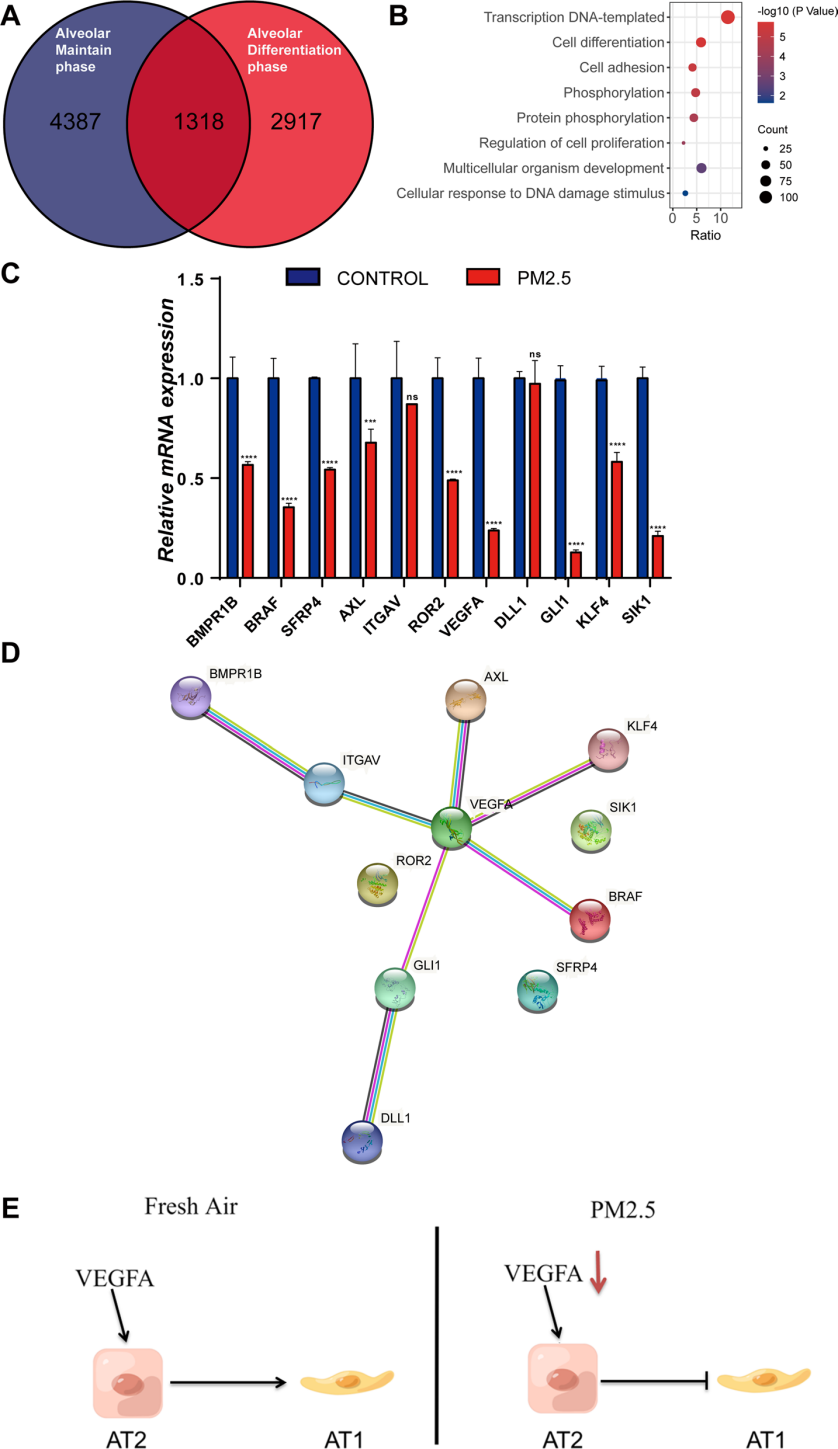
Down regulation of VEGFA may be the cause of abnormal AT2 damage and repair. **A** Venn diagram of down-regulated genes in alveolar maintain phase and alveolar differentiation phase. **B** GO analysis of intersecting down-regulated genes. **C** RT-qPCR was performed to verify down-regulated genes related to cell differentiation. **D** PPI analysis of down-regulated genes related to cell differentiation. **E** Hypothetical diagram of VEGFA regulating the differentiation process of AT2 cells. PM2.5 exposure reduced VEGFA expression leading to impaired AT2 to AT1 transition. ****p < 0.0001. Two-way ANOVA Ordinary. CONTROL vs PM2.5 exposure. Data presented are means ± SD. The experiments were repeated at least three times (n ≥ 3)

To understand the effect of PM2.5 exposure on alveolar damage and repair process, we performed RNA-sequencing analysis of alveolar organoids. Sequencing results showed that there were 5718 down-regulated genes and 2952 up-regulated genes in alveolar maintain phase (Fig. 4A), and 4261 down-regulated genes and 3969 up-regulated genes in alveolar differentiation phase. A complete gene list is provided in the supplementary document Additional files 1 and 2. KEGG analysis of the total 9979 down-regulated genes revealed that the associated pathways were enriched in Wnt signaling pathway, MAPK signaling pathway and signaling pathways regulating pluripotency of stem cells, which are related to the alveolar damage and repair process (Fig. 4B and E). GO analysis of down-regulated genes in alveolar maintain phase suggested that PM2.5 exposure influenced wound healing, response to wounding and epithelial cell migration processes (Fig. 4C). GO analysis of down-regulated genes in alveolar differentiation phase discovered that PM2.5 exposure effected stem cell differentiation and organ growth processes (Fig. 4F). Taken together, PM2.5 exposure might influence the repair of alveolar damage by down-regulation of related signaling pathways. In order to explore the specific genes that participated in PM2.5-induced abnormal alveolar damage-repair process, we performed the intersection of down-regulated genes and identified 1318 genes that were down-regulated in both phases (Additional file 3 and Fig. 5A). GO analysis of the 1318 genes showed that most of these genes were enriched in cell differentiation process (Fig. 5B). Validation of these specific genes was performed by RT-qPCR and reduced mRNA expression of BMPR1B, BRAF, SFRP4, AXL, ROR2, VEGFA, GLI1, KLF4 and SIK1 was observed (Fig. 5C), which is consistent with our RNA sequencing result (Additional file 3). A protein–protein interaction network analysis of these validated genes revealed that VEGFA was the most important gene (Fig. 5D). VEGFA has been identified as one of the most important cytokines in vascular and alveolar epithelial homeostasis [33]. Our results show that VEGFA is likely to play an essential role in the process of abnormal AT2 cell damage and repair induced by PM2.5 (Fig. 5E).

## Discussion

A number of previous studies have demonstrated an association between PM2.5 and the development of COPD [20–23], however the precise underlying mechanisms through which PM2.5 regulate the development of COPD remain largely unknown. In the present study, we investigated the effects of PM2.5 on AT2 to AT1 transition during COPD using animal models and in vitro organoids culture. We demonstrated that PM2.5 exposure significantly induced alveolar destruction in the alveoli of mice. Interestingly, PM2.5 exposure dramatically altered the ability of AT2 to AT1 transition. Through RNA-sequencing, we showed that PM2.5 exposure significantly altered several key signaling pathways involved in cell differentiation including Wnt signaling, MAPK signaling and signaling pathways regulating pluripotency of stem cells. These results advance our current understanding of the underlying mechanisms through which PM2.5 induce COPD, and may lead to a novel therapeutic strategy to treat COPD.

In this study, we found that long-term exposure of PM2.5 resulted in a significant decrease in pulmonary compliance and forced pulmonary ventilation in mice. This result is consistent with previous studies reporting that the dynamic lung compliance is decreased in COPD [34, 35]. In accordance with these observations, we found that PM2.5 exposure induced mouse pulmonary emphysema and reduced the mean number of alveoli in the mouse lung. These results are consistent with previous studies reporting that PM2.5 induced inflammation and tissue damages in the mouse lung [36–38]. During lung injury, AT1 cells are more sensitive to injury. Previous studies have demonstrated that AT2 cells are the primary cells to regenerate the injured alveolar epithelium [39, 40]. This function is primarily dependent on the capacity of AT2 cells for their self-renewal and differentiation to AT1 cells [41]. Interestingly, our in vitro and in vivo data showed that PM2.5 exposure significantly reduced the AT2 to AT1 cell transition in the mouse lung, thereby leading to the development of mouse COPD. However, we cannot exclude the possibility that whether AT2 to AT1 transdifferention also contributes to normal alveolar regeneration in the mouse lung repair which is induced by short-term PM2.5 exposure. It would be interesting to confirm this possibility using lineage tracing experiments in the future. To the best of our knowledge, this is the first report showing the dysregulated AT2 to AT1 cell transition in PM2.5-induced COPD mouse model. To date, it remains unknown whether transformation and differentiation from AT2 to AT1 is involved in the cigarette smoke-induced COPD mouse model. It would be interesting to investigate the differences in the gene expression profiles of murine AT2 cells during lung repair process between the PM2.5- and cigarette smoke-induced COPD mouse model.

Through RNA-sequencing, we observed that PM2.5 exposure significantly altered gene expression profile in mouse alveolar organoids. Bioinformatical analysis further demonstrated that the most significantly altered signaling pathways by PM2.5 exposure include Wnt signaling and MAPK signaling. Wnt signaling is evolutionarily conserved, and activation of this pathway leads to accumulation of beta-catenin in the nucleus to promote its target gene expression. Consistent with our results, previous studies have demonstrated the crucial role of Wnt signaling in AT2 to AT1 cell transition in the mouse lung [42, 43]. In addition, it has been reported that Wnt signaling plays a key role in regulation of cell proliferation, differentiation and migration during lung development [44]. Several key Wnt ligands such as Wnt5a, Wnt7b and most Frizzled receptors have been reported to be the important regulators of lung development [45–47]. Future studies would be interesting to investigate whether the expression of these Wnt ligands are altered during the development of PM2.5-induced COPD, and whether target of Wnt signaling could attenuate PM2.5-induced COPD.

MAPK signaling pathway plays an important role in cell proliferation, differentiation, cell survival and apoptosis. A previous study has reported that MAPK signaling pathway is required for normal lung development [48]. Interestingly, inhibition of MAPK signaling has been shown to protect lipopolysaccharide-induced lung injury [49]. Recently, Liu et al. has demonstrated that MAPK signaling is essential for promoting alveolar regeneration in response to mechanical tension in the lung [50]. Consistent with these studies, we showed that PM2.5 exposure significantly altered MAPK signaling pathway in mouse alveolar organoids culture. Our results extend on these previous studies and for the first time demonstrate a crucial role of MAPK signaling in PM2.5-induced lung damage and the development of COPD mouse model.

In addition, our RNA-sequencing and subsequent bioinformatical analysis revealed that VEGFA is the most significantly affected gene in alveolar organoids exposure to PM2.5. Our RT-qPCR confirmed that PM2.5 exposure significantly reduced VEGFA mRNA expression in mouse alveolar organoids. These data suggest that VEGFA signaling may play an important role in AT2 to AT1 cell transition and PM2.5-induced lung damage and repair. We showed that PM2.5 exposure significantly altered Wnt and MAPK signaling pathways, which are key regulators of VEGFA expression [51, 52]. Therefore, it is plausible to speculate that PM2.5 exposure downregulates VEGFA expression via altered Wnt and MAPK signaling pathways. Future studies would be interesting to address this possibility. In accordance with our results, previous studies have reported that reduced expression of VEGFA is associated with acute and chronic lung disease [53]. Moreover, chronic blockade of VEGF receptor has been shown to induce alveolar cell apoptosis and emphysema [54]. Future studies are warranted to investigate whether VEGFA supplementation could attenuate PM2.5-induced lung damage and impaired AT2 to AT1 transition.

## Conclusions

Overall, this study shows that long term PM2.5 exposure induced the development of emphysema and airflow limitation in mice, and reports for the first time a key role of AT2 to AT1 cell transition in PM2.5-induced COPD mouse model. Mechanistically, we report that PM2.5 exposure may reduce AT2 to AT1 cell transition in the mouse lung through regulation of Wnt signaling, MAPK signaling and VEGFA signaling. Taken together, this study further advances our current understanding of PM2.5-induced COPD, and may provide a novel therapeutic strategy for this disease.

## Supplementary Information

**Additional file 1. **Alveolar maintain phase RNA-seq, include RNA sequencing data of alveolar organoids in maintain phase.

**Additional file 2. **Alveolar differentiation phase RNA-seq, include RNA sequencing data of alveolar organoids in differentiation phase.

**Additional file 3. **List of 1318 genes, include gene names of 1318 genes that were down-regulated in maintain & differentiation phases in alveolar organoids after PM2.5 exposure.

**Additional file 4. **Supporting information, include supplementary figures. **Fig. S1.** (**A**) Schematic diagram of the construction of mouse alveolar organoids. (**B**) Quantitative of SPC^+^/total cells ratio per 20 × field, day 7 differentiating organoids. (**C**) Quantitative of HOPX^+^/total cells ratio per 20 × field, day 7 differentiating organoids. (**D**) Quantitative of HOPX^+^/total cells ratio at day 7 and day 17 in differentiating organoids exposed to PM2.5. **Fig. S2.** The validation of AT2 cells. (**A**) The purity of AT2 cells. Lungs from C57BL/6 mice were digested into a single-cell suspension and sorted by MicroBeads, the sorted cells were detected by CD326-APC. (**B**) Immunofluorescence staining of AT2 marker SPC and AT1 marker HOPX in CD45 negative CD326 positive cells.

## Data Availability

The datasets used and analyzed during the current study are available from the corresponding author on reasonable request.

## Abbreviations

PM2.5: Fine particulate matter
COPD: Chronic obstructive pulmonary disease
AT2: Alveolar type II cell
AT1: Alveolar type I cell
TGF: Transforming growth factor
FRC: Functional residual capacity
FV: Fast flow volume
PV: Quasi-static pressure volume
RC: Resistance and compliance
PAHs: Polycyclic aromatic hydrocarbons
SRM: Standard reference material
H&E: Haemotoxylin and Eosin
Cdyn: Dynamic lung compliance
FPKM: Fragments per kilobase million
DMSO: Dimethyl sulfoxide
CLDN4: Claudin 4
SPC: Pro-surfactant protein C
CLDN4: Claudin 4
MAPK: Mitogen-activated protein kinase
FBS: Fetal bovine serum
PBS: Phosphate buffered saline
EGF: Epidermal growth factor
FGF 10: Fibroblast growth factor 10
IL 1β: Interleukin 1 Beta
BSA: Bovine serum albumin
HOPX: HOP homeobox
Ki67: Marker of proliferation Ki-67
Gapdh: Glyceraldehyde-3-phosphate dehydrogenase
Cdc25c: Cell division cycle 25C
BMPR1B: Bone morphogenetic protein receptor type 1B
AXL: AXL receptor tyrosine kinase
VEGFA: Vascular endothelial growth factor A
KLF4: Kruppel like factor 4
BRAF: B-raf proto-oncogene, serine/threonine kinase
ROR2: Receptor tyrosine kinase like orphan receptor 2
SIK1: Salt inducible kinase 1
SFRP4: Secreted frizzled related protein 4
ITGAV: Integrin subunit alpha V
GLI1: GLI family zinc finger 1
DLL1: DLL1 delta like canonical Notch ligand 1
