# Supplementary material for: Impaired AT2 to AT1 cell transition in PM2.5-induced mouse model of chronic obstructive pulmonary disease
Source: Respir Res. 2022 Mar 25;23:70. doi: 10.1186/s12931-022-01996-w (PMC8957194; doi:10.1186/s12931-022-01996-w)
Supplement: Supplementary file 4 — Additional file 4. Supporting information, include supplementary figures. Fig. S1. (A) Schematic diagram of the construction of mouse alveolar organoids. (B) Quantitative of SPC+/total cells ratio per 20 × field, day 7 differentiating organoids. (C) Quantitative of HOPX+/total cells ratio per 20 × field, day 7 differentiating organoids. (D) Quantitative of HOPX+/total cells ratio at day 7 and day 17 in differentiating organoids exposed to PM2.5. Fig. S2. The validation of AT2 cells. (A) The purity of AT2 cells. Lungs from C57BL/6 mice were digested into a single-cell suspension and sorted by MicroBeads, the sorted cells were detected by CD326-APC. (B) Immunofluorescence staining of AT2 marker SPC and AT1 marker HOPX in CD45 negative CD326 positive cells. [file 12931_2022_1996_MOESM4_ESM.docx]

**Supporting information**

**Title：**Impaired AT2 to AT1 Cell Transition in PM2.5-induced Mouse model of Chronic Obstructive Pulmonary Disease

Hongjiao Yu^#1^, Yingnan Lin^#1^, Yue Zhong^#1^, Xiaolan Guo^1^, Yuyin Lin^1^, Siqi Yang^1^, Jinglin Liu^1^, Xinran Xie^1^, Yaowei Sun^1^, Dong Wang^2^, Jianwei Dai*^1,2^

1. Guangzhou Medical University-Guangzhou Institute of Biomedicine and Health (GMU-GIBH) Joint School of Life Sciences, Guangzhou Medical University, Guangzhou, 510000, China.

2. State Key Lab of Respiratory Disease, National Clinical Research Center for Respiratory Disease; Guangzhou Institute of Respiratory Disease, The First Affiliated Hospital of Guangzhou Medical University, Guangzhou 510120, China.

**This word file includes:**

Supplementary methods

Figure S1-S2

**Supplementary Methods**

**Validation of AT2 cells**

Validation of mouse AT2 cells was performed using flow cytometry analysis (Beckman, cytoflex S). Single-cell suspensions of murine lung tissue were stained and gated based on the following criteria: positive for CD326-APC (BioLegend; G8.8) and negative for CD45-FITC (BioLegend; 103108).

**Supporting Figures**

**Figure S1**

**
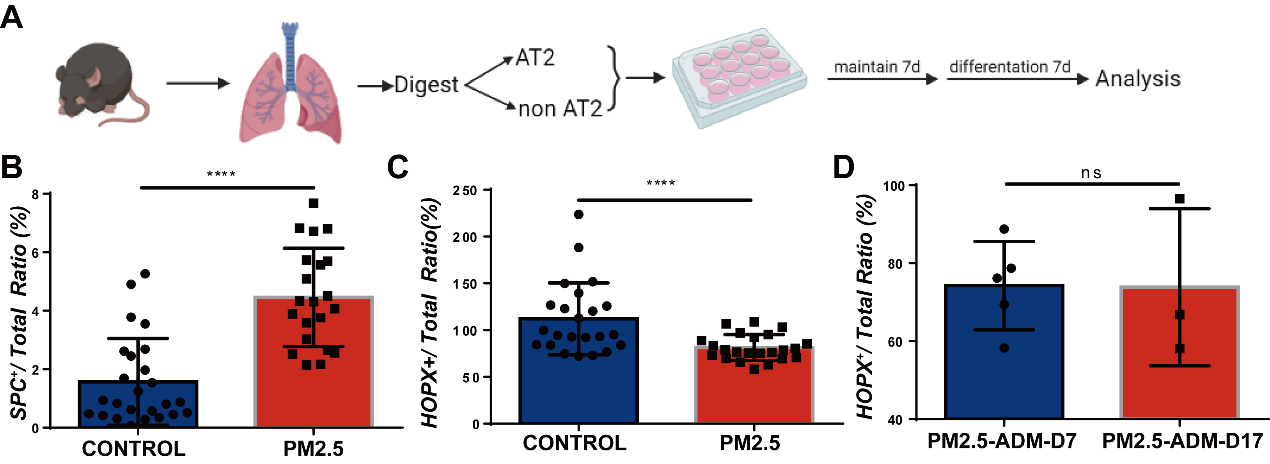
**

**Fig. S1***.* (**A**) Schematic diagram of the construction of mouse alveolar organoids. (**B**) Quantitative of SPC^+^/total cells ratio per 20x filed, day 7 differentiating organoids. (**C**) Quantitative of HOPX^+^/total cells ratio per 20x filed, day 7 differentiating organoids. (**D**) Quantitative of HOPX^+^/total cells ratio at day 7 and day 17 in differentiating organoids exposed to PM2.5.

**Figure S2**

**
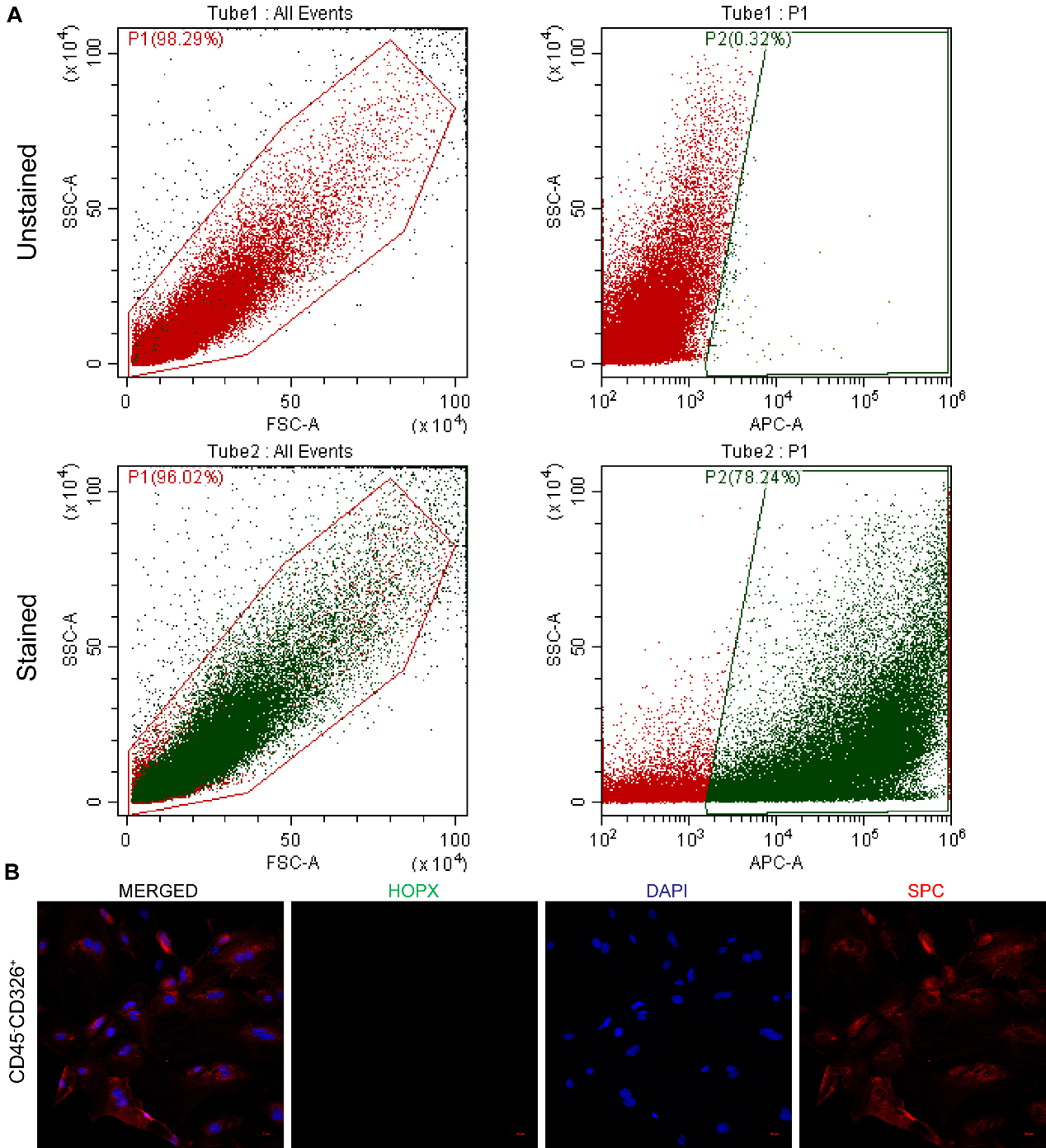
**

**Fig. S2***.* (**A**) The purity of AT2 cells. Lungs from C57BL/6 mice were digested into a single-cell suspension and sorted by MicroBeads, the sorted cells were detected by CD326-APC. (**B**) Immunofluorescence staining of AT2 marker SPC and AT1 marker HOPX in CD45 negative CD326 positive cells.
